# Editorial: Transdisciplinary Research on Learning and Teaching: Chances and Challenges

**DOI:** 10.3389/fpsyg.2021.696219

**Published:** 2021-07-06

**Authors:** Matthias Stadler, Arthur Graesser, Frank Fischer

**Affiliations:** ^1^Department of Psychology, Ludwig-Maximilians-Universität Munich, Munich, Germany; ^2^Department of Psychology and the Institute for Intelligent Systems, University of Memphis, Memphis, TN, United States

**Keywords:** interdisciplinary, transdisciplinary, Education, teachers, methods

## Introduction

The goal of the present Research Topic is to provide a forum where research groups, investigating teaching and teachers from multiple perspectives involving multidisciplinary (i.e., different disciplines working on different aspects of a problem independently within their disciplinary boundaries), interdisciplinary (i.e., restructuring and integrating existing disciplinary approaches to address problems relevant for all participating disciplines) and ideally transdisciplinary (i.e., seeking to integrate different lines of work from contributing disciplines to create new approaches or even new scientific disciplines) approaches (Klein, 2017; Hall et al., 2018), can present and discuss the opportunities and challenges of such endeavors. The articles published in the Research Topic can be broadly classified into three categories: Conceptual reviews of transdisciplinary research on teaching and teachers, the results of transdisciplinary research projects, and methodological challenges and innovations related to transdisciplinary cooperation.

## Conceptual Reviews of Transdisciplinary Research

The Research Topic is initiated by Pea and Linn, who provide their personal perspectives on the emergence of the learning sciences as a transdisciplinary research community from the early 1970's to today. In line with the Research Topic's aim, the paper illustrates how the specific approach of the learning sciences integrated approaches from disciplines as diverse as science education, psychology, and computer science to create a new and more holistic scientific discipline devoted to research on learning and instruction under a situated cognition perspective.

Their article is complemented by Lund et al., who discuss how research in education draws widely from the social sciences and humanities. The study uses bibliometric analyses to determine the place of educational research in the larger context of social science research. The authors argue that modern educational research cannot be considered to be a single discipline but rather a multidisciplinary field, thus implementing the initial goal described by Pea and Linn.

This interdisciplinarity of modern educational research is also mirrored in Hmelo-Silver and Jeong's review on the benefits and challenges of interdisciplinarity in computer-supported collaborative learning, a research field remarkably diverse regarding contributing disciplines. While the authors agree that diversity should be cultivated, they also caution to be mindful that research outcomes need to be exchanged and appropriated actively across participating disciplines in order for our understanding of CSCL rises above individual disciplines.

Two articles discussing student learning and development in university settings as a systematic way to integrate different academic disciplines complete the section. Budwig and Alexander take a firm stance for reorganizing universities and curricula on campus efforts to allow disciplinary integration that requires not only alignment and support from the learning and developmental sciences but also local, national, and transnational efforts with relevant learning communities.

Pammer-Schindler et al. present their experiences with interdisciplinary doctoral training on technology-enhanced learning (TEL) in Europe. Based on a survey of 35 doctoral education programs in Europe, the authors argue that cross-institutional doctoral training might be key to progress TEL as a field.

While these challenges are significant, all five articles in this section show how much progress was made in the last decades toward transdisciplinary research on teaching and teachers.

## Results of Interdisciplinary Research Projects

The second section of this Research Topic takes a step back from the theoretical challenges of transdisciplinary research to illustrate concrete lessons learned from current transdisciplinary research projects.

Schilcher et al. detail how the FALKE (Fachspezifische Lehrerkompetenzen im Erklären; Engl.: subject-specific teacher competency in explaining) research project integrates 14 heterogeneous disciplines in order to examine the pedagogical quality of teacher explanations empirically. The authors discuss how trans-, multi-, and interdisciplinary projects, in particular, are primarily shaped by the nature of the problem, the scientists and stakeholders involved, and the institutional setting. Moreover, they present an example on how to tackle some of these issues.

Closely related to this, Heitzmann et al. illustrate the potential but also the challenges of large transdisciplinary projects. The authors review why many promising projects fail beyond the actual research conducted. They argue that ideas from the field of collaborative problem solving have the potential to yield valuable insights when designing or conducting cross-disciplinary research in learning and instruction.

Bauer et al. present an innovative analytic approach based on epistemic network analysis to compare diagnostic activities in medical and teacher education. Based on their results, the authors recommend that educators think beyond individuals' knowledge and systematically teach and increase the awareness of disciplinary standards.

Finally, Fleckenstein et al. investigate whether text length is a construct-relevant aspect of writing competence, a transdisciplinary issue concerning the research areas of educational assessment, language technology, and classroom instruction.

All articles in this section provide examples of successful interdisciplinary research projects and highlight the challenges that come with such endeavors.

## Methodological Challenges and Innovations

The final part of this Research Topic focuses on the methodological challenges posed by transdisciplinary research. Lindl et al. provide recommendations on tackling the often highly complex data resulting from investigating unique subject-specific aspects on the one hand and transdisciplinary, generalizable effects on the other. They compare meta-analysis, multilevel models, latent multilevel structural equation models, and machine learning methods discussing the advantages and disadvantages of all methods.

Levy et al. contrast classical and machine learning approaches in estimating value-added scores in large-scale educational data. Aside from statistical features, the authors discuss possible ethical concerns and practical implications regarding using machine learning methods for decision-making in education.

The Research Topic is concluded by Rienties et al. The authors review future research directions on teaching and teacher education, defining the boundaries between artificial intelligence in education, computer-supported collaborative learning, educational data mining, and learning analytics. The article encourages researchers to cross the boundaries of their respective fields and work together to address the complex challenges in education.

In this collection, we included meta-level and theoretical papers on collaborations between various disciplines in research on learning, the design of learning environments, and teaching. These approaches can serve as models for future collaborations to tackle complex phenomena and problems that are beyond what individual disciplines can tackle successfully.

## Author Contributions

All authors listed have made a substantial, direct and intellectual contribution to the work, and approved it for publication.

## Conflict of Interest

The authors declare that the research was conducted in the absence of any commercial or financial relationships that could be construed as a potential conflict of interest.
